# Diagnostic Accuracy of CBCT for Detection of Second Canal of Permanent Teeth: A Systematic Review and Meta-Analysis

**DOI:** 10.1155/2021/1107471

**Published:** 2021-07-20

**Authors:** Nyan M. Aung, Kyaw K. Myint

**Affiliations:** Department of Oral Biological Science, University of Dental Medicine, Mandalay 05041, Myanmar

## Abstract

**Introduction:**

Missed canal is one of the common reasons for nonsurgical endodontic retreatments. The missed canals were frequently associated with periapical pathology. The aim of this systematic review was to find the diagnostic accuracy of CBCT for detection of the second canal of the root canal system of permanent teeth.

**Materials and Methods:**

The articles were selected from seven electronic databases according to selection criteria. All eligible studies were judged by the reviewers. The selected studies were checked with the QUADAS-2 tool for risk of bias and applicability concerns. Finally, 12 studies were selected for qualitative and quantitative analyses. The summary estimates of sensitivities and specificities and SROC curves were calculated and drawn by RevMan 5.3 and MetaDTA software.

**Results:**

Summary estimates of CBCT for detection of second canal anatomy in permanent teeth were 94% sensitivity and 93.1% specificity. 96.6% sensitivity of MB2 was followed by 88.8% sensitivity of maxillary and mandibular premolars and 81% that of mandibular molars. The specificity of 97.6% for premolars was trialed by 85% specificity of mandibular molars and MB2. For permanent mandibular canines, 67% sensitivity and 100% specificity were estimated. CBCT showed more agreement with detecting the second canal with micro-CT, estimating 100% sensitivity and 95.6% specificity. The highest prevalence of the second canal comprised the highest sensitivity of 99.1% and lowest specificity of 77.5%. After the exclusion of case-control studies, a 3% drop of sensitivity from the summary estimate was observed. Multiple spectrum of the second canal had 8.6% higher sensitivity and 4.4% lower specificity than single spectrum.

**Conclusion:**

CBCT is informative for detecting the second canal. Clinicians should keep in mind that the accuracy can vary in different types of teeth, with the prevalence of second canal across different populations, and with the spectrum of second canal anatomy in spite of the reviewers having postulated overestimation of the findings.

## 1. Introduction

The term “diagnosis” is derived from “di-ac-ri-sis” [1], a prehistoric Greek word, actually meaning “knowing through” [2]. It is more important than treatment. False results in diagnosis can be false negative and false positive. False-negative results lead to delay in proper management, increasing morbidity of disease and leading to a more invasive intervention. False-positive results lead to unnecessary treatment and follow-up.

Diagnosis of root canal anatomy is a routine measure of current dental practice. Plenty of systematic reviews have revealed pooled estimates of the prevalence of root canal anatomy [3–6]. Of these, the investigation of the second canal is implicated in both clinical and research scenarios.

One previous systematic review [5] has investigated that the pooled estimates of the prevalence of the second canal in permanent mandibular central and lateral incisors were more than 5% and 14%, respectively, in the Chinese population. The prevalence of the second canal of maxillary first premolar ranged from 37% to 97% [7, 8]. This anatomy of mandibular first premolar ranged from 6% to 23% [7, 8]. The summary estimate of 21 studies found that 60% prevalence of MB2 was detected in mesiobuccal roots of maxillary first molars [9]. This anatomy of the mesial root of mandibular first molars was present in 94% of the study population in one systematic review [6].

As a result, the diagnosis of the second canal can be correctly identified to some extent through CBCT scanning. The sensitivity of the CBCT in detecting the type of root canal system was estimated to be 79% compared to that of micro-CT. One study has pointed out that the agreement between CBCT and micro-CT was 88.6%. In routine dental practice, visual inspection, Dental Operating Microscope (DOM), and digital X-ray [10] were preliminary tests for detection of the second root canal. CBCT is usually used as an add-on test after these initial assessments. In spite of having a longer scanning time to achieve a better resolution and a questionable sensitivity, MRI (Magnetic Resonance Imaging) can also be used to identify root canal anatomy because it can be readily applicable to clinical and in vivo situations, also by avoiding radiation [11].

The optimal diagnosis and proper endodontic management can fulfill the survival of the tooth. One systematic review has pointed out that eight to ten years of survival was seen in the tooth, which had better prognostic indicators, treated by conventional root canal therapy [12]. Thus, diagnostic imaging is crucial in endodontic treatment. If a second canal in permanent teeth can be detected prior to endodontic treatment, then general dental practitioners (GDP) and endodontists can avoid unnecessary endodontic treatment failure, undertake timely referral, and can reduce high costs for the patients. CBCT is useful in vivo and clinical practice, has a lower radiation dose and processing time than micro-CT, and can be applicable in epidemiological settings [13].

On the other hand, lack of diagnostic ability results in over- and undertreatment. Missed second canal was the fourth most common endodontic failure, which needs retreatment [14]. The missed canals were frequently associated with periapical pathology [15]. Vertucci's Type Ι root canal (one canal and one foramen) was misdiagnosed in CBCT as Type ΙΙΙ [16], false-positive second canal in permanent mandibular first premolar. Additionally, Type Ι of the premolar was misinterpreted as Type VΙ [16], false-positive second canal in CBCT scan. Moreover, Types ΙV and V (second canal) of human premolars were wrongly screened as Type Ι, false-negative second canal in CBCT [17]. In summary, root canal anatomy can be misdiagnosed in CBCT images.

Additionally, the sensitivities of CBCT for detection of apical delta and lateral canal were 35% and 55%, respectively [18, 19]. So, CBCT has limited ability in investigating some features of root canal anatomy. However, the European Society of Endodontology recommended that the teeth with complex root canal anatomy, which will undergo nonsurgical endodontic retreatment, were eligible for CBCT with limited field of view (FOV) [20].

To increase the visibility of root canal anatomy, reduced voxel size and increase in scanning time were the main options of high-resolution mode, although milliampere, FOV, kilovolt, and slice thickness should also be considered [21]. However, improper utilization of these factors may potentially increase radiation dose. Of these, smaller FOV reduces radiation exposure to patients and increases CBCT image resolution and was preferred in investigating endodontic anatomy [21]. Contrastively, FOV was desirable to be large enough to include all of the maxillofacial regions in the scope of orthodontic diagnosis to investigate soft tissue facial profiles and some of the facial anatomical landmarks [22].

Advertising of manufacturers of medical devices, the competitive business practice [23], industrial sponsorship, and individual conflict of interests in research practice may contribute to overtreatment. However, the actual benefit of these devices, including CBCT, is still questionable. Because of the financial burden and radiation dose, CBCT should be used cautiously for patient safety and benefit.

So, the cost and benefits of usage of CBCT should be taken into consideration before proceeding with endodontic treatment. In research, DTA (Diagnostic Test Accuracy) studies had a small sample size [16, 17], which was not sufficiently generalizable to the target population.

As a result, the diagnostic accuracy of CBCT in detecting the second canal was needed to be systematically reviewed and meta-analyzed to obtain more generalizable findings and more precise pooled estimates. Besides the above-mentioned reviews, no systematic reviews of DTA (Diagnostic Test Accuracy) studies are available in this era.

The research question of this systematic review was as follows: what is the diagnostic accuracy of CBCT for detection of the second canal of the root canal system of permanent teeth?

The primary objective was as follows:

1. To investigate the summary estimates of the accuracy of CBCT for detection of the second canal of permanent teeth

 To explore heterogeneity, the secondary objectives were as follows:

1. To examine the accuracy of CBCT for detection of the second canal of permanent teeth according to different types of teeth
2. To evaluate the accuracy of CBCT for detection of the second canal of permanent teeth according to different reference standards
3. To identify the accuracy of CBCT for detection of the second canal of permanent teeth according to levels of prevalence
4. To estimate the summary accuracy of CBCT for detection of the second canal of permanent teeth after exclusion of diagnostic case-control studies
5. To observe the accuracy of CBCT for detection of second canal of permanent teeth according to single and multiple spectra of the second canal

## 2. Materials and Methods

### 2.1. Selection Criteria

#### 2.1.1. Types of Studies

DTA (diagnostic test accuracy) studies, which were eligible for selection, were as follows:

1. Diagnostic cohort studies: the outcome of interest (second canal) of the sampled teeth was not a priori known as case (presence of second canal) and control group (absence of second canal). All sampled teeth passed through both index test (CBCT) and reference standard or comparison test (micro-CT, staining and clearing, or root sectioning). This was called “cohort” meaning “marching together.”
2. Diagnostic case-control studies: the outcome of interest (second canal) of the sampled teeth was initially known before the conduction of the study. Case (second canal) and control were detected and selected a priori by negotiating #10 K-File or DOM (Dental Operating Microscope) or by intervening reference standard first.
3. Agreement study: this is matched with the design of diagnostic cohort study and had the available data for calculation of a 2 × 2 contingency table.
4. In vitro study: this was included because the reference standard test (micro-CT or staining and clearing or root sectioning) of DTA studies of CBCT in detecting root canal anatomy cannot be conducted in vivo.

When the multiple observations of a single examiner were present, the first observation was selected for data extraction. When multiple examiners provided many observations on the index test, the observation of the general dental practitioner or endodontist was eligible to extract the available data. When the observation of the two oral healthcare professionals was not available, that of the oral radiologist was preferred. When the two observers had the same academic qualification, the observation of the first one was selected. The observation of undergraduates or postgraduates dental students was not eligible.

### 2.2. Characteristics of Excluded Studies

The exclusion criteria of the studies were as follows: data that were impossible to construct in a 2-by-2 contingency table, index test or comparison test not being CBCT, having none of the eligible reference standards, diagnosis of deciduous teeth, diagnosis of external morphology, diagnosis of calcified canal, unit of analysis being tooth section, the accuracy of CBCT for detection of intricate root canal anatomy and impaired data with two “0” cells because MetaDTA software does not allow two “0” cells for construction of a 2-by-2 table.

Editorials, case reports, systematic reviews, narrative reviews, opinions, book chapters, personnel communication, and letters to the editor were also excluded.

### 2.3. Population

Permanent teeth with no root deformity, nonendodontically treated prior to conducting the study, and no external or internal resorption were eligible.

Exceptionally, the study, in which the crown was sectioned and the main root canal was prepared and filled with gutta-percha also with no regard for coronal restoration and after starting the study, was selected to increase the number of studies in the meta-analysis, thereby improving statistical power and reducing the chance of beta error. In this type of study, the second canal, such as MB2, was intentionally unprepared and unfilled to resemble a “missed second canal.”

On the one hand, this type of study was similar to the real-life scenario, although beam hardening artifact (image scattering) was adjusted in CBCT image. On the other hand, the study, which was undergone with the teeth having coronal restorations and posts, was excluded in this review.

The unit of measurement was the root, not the tooth or patient. Single-rooted tooth was counted as “root” except for human premolar or canine which was counted as one root regardless of single or multiple roots. The roots of multirooted teeth were enumerated separately.

### 2.4. Index Test

The index test was CBCT. When multiple comparisons of different types of CBCT scans were undertaken in the selected study, the commonly used model was selected. Other comparison tests, such as X-ray, Dental Operating Microscope, visual inspection and Loupes, were not considered.

### 2.5. Reference Standard

The eligible reference standards were micro-CT, staining and clearing, and root sectioning. The studies, in which two or three reference standards were compared in spite of the index test not being included, were not selected. When the two reference standards were used in the same study, the more current method was selected (when micro-CT versus staining and clearing, micro-CT was eligible).

### 2.6. Target Condition

#### 2.6.1. Second Canal

Vertucci's classification [24] for root canal anatomy is as follows:

1. Type I is one canal from pulp cavity to the root apex (1-1 configuration).
2. The outcome of interest of this systematic review was the second canal which included other types of Vertucci's classification except Type I: Type II, two canals having left the pulp cavity but uniting near the apex to drain as a single foramen (2-1 configuration); Type III, one canal that diverges into two and then unite to drain as one (1-2-1 configuration); Type IV, separated two canals from entrance to the apex (2 configuration); Type V, one canal from the orifice and dividing two canals at the apex (1-2 configuration); Type VI, 2 canals that unite and are redivided into 2 canals (2-1-2 configuration); Type VII, one canal that separates, unites, and drains as two canals out of the root (1-2-1-2 configuration); Type VΙΙΙ, three separated canals; Type ΙΧ, three canals, uniting and piercing into one foramen at the external surface of the root (3-1 configuration); Type Χ, two canals uniting into one, then dividing as two, and then reuniting into one canal at the apex (2-1-2-1 configuration). In this systematic review, Types ΙΙ, ΙΙΙ, ΙV, V, VΙ, VΙΙ, VΙΙΙ, ΙΧ, and Χ were amalgamated as second canal (the target condition). More precisely, types of Vertucci's classification other than Type Ι were stated as second canal.

When the original study used different classifications other than Vertucci's, more than one canal was set as second canal. However, the second canal anatomy, which was not identified clearly in the primary study, was excluded from the sample.

Importantly, the second canal, which was misclassified as another type of the second canal in the primary study, was counted as true positive. Especially, the second canal, which was erroneously identified as one canal and one foramen (TypeΙ), was set as a false negative.

#### 2.6.2. Spectrum of the Second Canal

There are two spectrum types as follows:

1. Single spectrum of second canal: the sampled teeth had a single type of second canal anatomy such as only Vertucci's Type ΙΙΙ.
2. Multiple spectrum of second canal: the sampled teeth had multiple types of second canal such as Vertucci's Types ΙΙ, ΙΙΙ, ΙV, V, VΙ, VΙΙ, VΙΙΙ, ΙΧ, and Χ and other categories.

#### 2.6.3. Search Strategies

The literature search was done with the breakdown of the research question: population (P), index test (I), reference standard or comparators or comparison (C), and target condition or outcome of interest (O) (PICO).

Search terms were “Permanent Teeth,” “Root canal,” “Root canal anatomy,” “Second Canal,” “Second Root Canal,” “MB2,” “Cone-beam computed tomography,” “CBCT,” “micro CT,” “*µ* CT,” “Staining and Clearing,” “Diaphanization,” “Tooth Clearing,” “Root Sectioning,” “Tooth Sectioning,” “Comparative,” “Comparison,” “diagnostic,” “diagnosis,” “sensitivity,” “specificity,” “Accuracy,” “Receiver Operator Curve,” “Receiver Operating Characteristic Curve,” “ROC curve,” “Positive Predictive Value,” “PPV,” “Negative Predictive Value,” “NPV,” “Diagnostic Odds Ratio,” “DOR,” “Positive Likelihood Ratio,” “Negative Likelihood Ratio,” “Area Under the Curve,” and “AUC.”

The search strategies were set through the following: (1) “Index Test (CBCT)” AND “Target Condition (Second Root Canal, MB2, and Root Canal Anatomy)”; (2) “Target Condition” AND “Reference Standard (micro-CT, Staining and Clearing, and Root Sectioning)”; (3) “Index Test” AND “Diagnostic Test Accuracy Terms (diagnostic, diagnosis, sensitivity, specificity, Accuracy, ROC curve, Receiver Operator Curve, Receiver Operating Characteristic Curve, Positive Predictive Value, PPV, Negative Predictive Value, NPV, Diagnostic Odds Ratio, DOR, Positive Likelihood Ratio, Negative Likelihood Ratio, Area Under the Curve, and AUC); (4) 1 OR 2 OR 3.

The databases, which were used for literature search for this systematic review, were PubMed, Goggle Scholar, Research Gate, Hinari “Research4Life,” LILACS, and for grey literature, conference paper, theses and dissertations, ProQuest and Scopus were searched. Language matter was restricted to English. Snowballing from the references of the articles was done. Searching was performed from 1^st^ June 2020 to 29^th^ June 2020. The papers were published between 1^st^ Jan 2010 to 31^st^ Dec 2019. The opinion from the librarian (information specialist) of the University of Dental Medicine (Mandalay), Myanmar, was taken during the literature search. The steps of selecting articles were presented with PRISMA (Preferred Reporting Items for Systematic Reviews and Meta-Analyses) Flow Diagram [25].

### 2.7. Data Collection

One review author (NMA) selected the eligible studies independently. Another reviewer (KKM) checked the selected studies. The disagreement, which was present between the two reviewers, was resolved by discussion until the consensus was reached.

We recorded the following data for each study: sample characteristics (number of teeth, number of roots, types of teeth, prevalence of second canals, and spectrum of second canal), setting (country and type of simulated environment or artificial setting), types of index test used (brand and model of CBCT, Field of View (FOV), voxel size, milliampere (mA), processing time (seconds), slice thickness, and kV), study information (types of study design, types of reference standard, calibration of observers, number of observer, and types of observers), and study results (true positive, true negative, false positive, false negative, and drop-out). When some studies reported the proportions of study findings, the results were calculated retrospectively to obtain a 2 × 2 table.

### 2.8. Assessment of Methodological Quality

We used Quality Assessment of Diagnostic Accuracy Studies 2 (QUADAS-2) to investigate the risk of bias and applicability concerns of selected primary studies according to the four domains: participant selection, index test, reference standard, and flow and timing [26]. However, the applicability concerns were identified through only three domains such as patient selection, index test, and reference standard. The assessments of risk of bias and applicability concerns were described in the graphics. The risk of bias can be concerned with and can influence the findings of the meta-analysis. By correlating with the risk of bias of primary studies, the reviewers can correlate the findings of the meta-analysis with the risk of bias cautiously. The applicability concerns were related to whether the setting of primary study was applicable or not to the clinical setting.

### 2.9. Statistical Analysis

First of all, the individual sensitivity and specificity of the eligible studies were calculated in RevMan 5.3. Then, the forest plots were created with these data. The summary estimates of sensitivity and specificities were calculated using a bivariate hierarchical model [27].

The resulting data from the individual study was entered into an example of an Excel spreadsheet of “CSV” file downloaded from the Shiny Website of MetaDTA https://crsu.shinyapps.io/dta_ma/, web-based software. After this, the data of the selected studies were deposited into the downloaded file. These data were ID, author name, year, TP (True Positive), FP (false positive), FN (false negative), and TN (True Negative). In this review, the covariate data were types of teeth, types of the reference standard, level of prevalence, types of study design, and spectrum of second canal. These variables were also entered into the Excel spreadsheet of “CSV” file.

The created “CSV” file was uploaded to the Shiny MetaDTA website. Then, random effect meta-analysis and sensitivity analyses were performed on this website using bivariate hierarchical model [27]. Finally, the results were downloaded from the site. The important results downloaded were summary sensitivity and summary specificity with a 95% confidence interval. The other downloaded data were logit sensitivity, logit specificity, variance of logit sensitivity, variance of logit specificity, logit correlation, standard error of logit sensitivity, standard error of logit specificity, and covariates estimate, which were in turn entered into the RevMan 5.3 to draw SROC (summary receiver operating characteristic) curve. These were also called the parameters for RevMan (“CSV” file).

The resulting summary estimates of sensitivity and specificity with the pooled prevalence of included studies were entered into a hypothetical cohort of 1000 roots to become the understandable findings for the audience.

The sensitivity analyses according to types of teeth, types of the reference standard, level of prevalence, types of study design, and types of spectrum of second canal were presented. The pooled estimates of sensitivities and specificities and SROC curves according to individual sensitivity analysis were calculated as mentioned above.

Within-study heterogeneity was described as confidence region and between-study heterogeneity was drawn as prediction region in the SROC curve. The confidence region and prediction regions can vary with the a priori defined covariates.

### 2.10. Publication Bias Method

To detect whether a small study has a large effect (overestimation) or not, DORs (Diagnostic Odds Ratio) with 95% confidence interval of primary studies were transformed to logarithmic Diagnostic Odds Ratio (lnDOR) with Standard Error (SE). After there, a dataset of lnDOR and SE of all included studies was exported into JASP 0.8.4.0 software to undergo trim-and-fill method [28], publication bias method. At least ten selected studies were needed for the publication bias method.

## 3. Results

**Figure 1:**
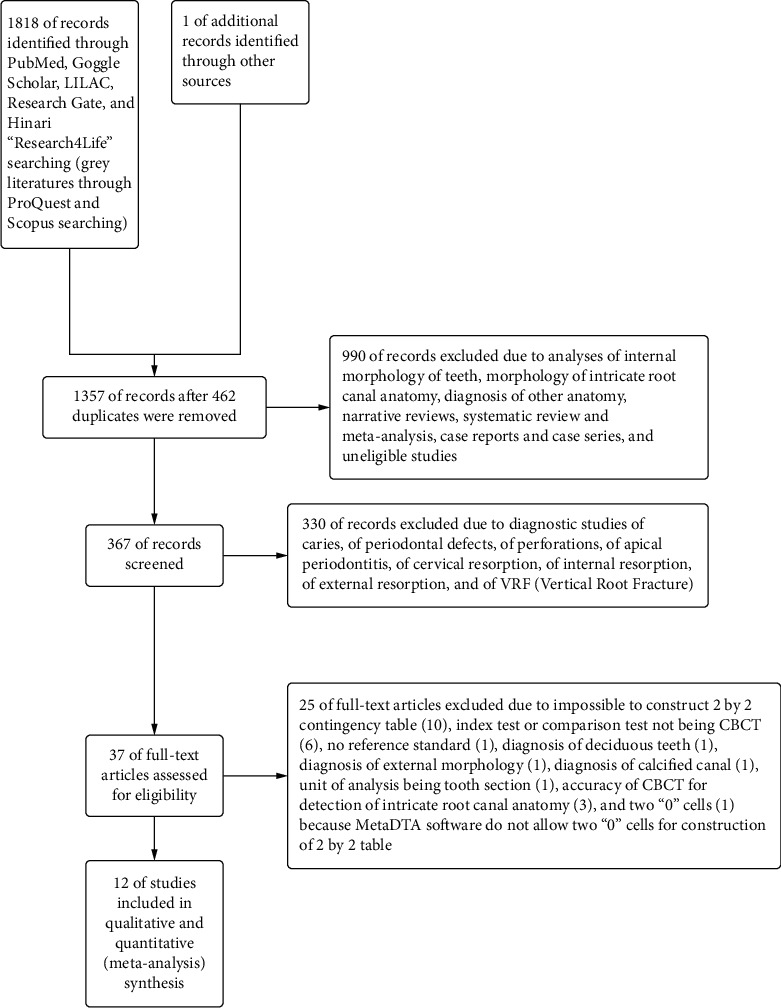
PRISMA flow diagram of the included and excluded studies.

Steps of screening and filtering the literature were demonstrated in PRISMA (Preferred Reporting Items for Systematic Reviews and Meta-Analyses) flow diagram (see Figure 1).

Twelve eligible studies [16, 17, 21, 29–37] were included for both qualitative and quantitative analyses (meta-analysis).

Furthermore, three studies investigated both second canal and intricate root canal anatomy. Of these three records, one identified both second canal and apical delta [29], one investigated both second canal and isthmus [30], and one assessed both second canal and lateral canal [31].

One study was undertaken by filling the root of the sampled teeth [32].

All 12 studies that were identified as providing diagnostic accuracy of CBCT for detection of second canal anatomy were published from 2010 to 2019.

Of 12 included studies, five were from Brazil [17, 21, 30, 33, 34] and three from Iran [29, 31, 35] and one was from China [16], 1 from USA [36], 1 from The Netherlands [32], and one from Malaysia [37].

**Figure 2:**
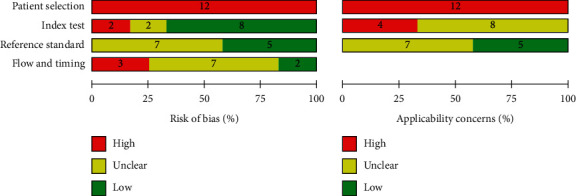
Risk of bias and applicability concerns graph for the included studies (review authors' judgments about each domain presented as percentages across included studies).

**Figure 3:**
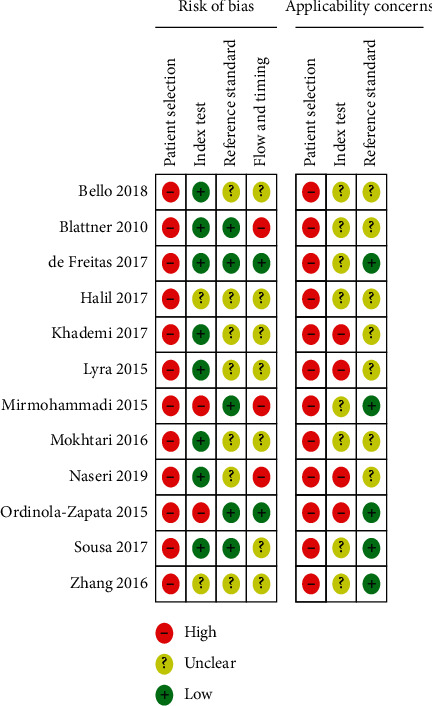
Risk of bias and applicability concerns summary for the included studies.

Risk of bias and applicability concerns graphs of 12 included studies were presented (see Figure 2). Risk of bias and applicability concerns summary of 12 included studies were appraised (see Figure 3).

For the patient selection domain, all of the included studies were stated as high risk of bias due to lack of randomization or consecutive series. All of the studies used the convenience sampling method, nonprobability sampling.

For the patient selection domain of applicability concern, all selected studies were in vitro studies. As a result, the in vitro design cannot be entirely applicable to the real clinical setting.

For the domain of index test, two [32, 33] of the included studies were stated as high risk of bias because the results of the index test (CBCT) were interpreted with the knowledge of reference standard (micro-CT). Two studies [16, 37] were scored as unclear risk of bias because the finding of the index test (CBCT) was interpreted and not clearly stated whether with or without the knowledge of reference standard.

For the index test domain of the applicability concern, simulated or artificial environment with the sampled teeth (periodontal ligament, alveolar bone, and gingiva) was not used in four selected studies [29–31, 35] during CBCT scan. This scenario cannot be applicable to the real-life clinical situation. The remaining eight studies [16, 17, 21, 32–34, 36, 37] were undergone with dry mandible, pig maxilla, pig mandible, equal part of gypsum and rice flour, Plaster of Paris (POP), resin block, and condensation silicone in tray used as alveolar bone, water and wax as gingiva and modeling, or utility wax as periodontal ligament (PDL) as the artificial environment during CBCT scan. So, the authors cannot clearly identify whether these settings are similar or not to the clinical setting.

For the reference standard domain of risk of bias, the reviewers affirmed that the findings of the reference standard of seven included studies [16, 29–31, 34, 35, 37] were not clearly interpreted whether with or without knowledge of finding of the index test (CBCT).

For the reference standard domain of applicability concern, seven studies [29–31, 34–37] included root sectioning and staining and clearing methods. These methods were not clearly applicable to the current research practice as gold standard methods and may have man-made errors.

Three of the selected studies [29, 32, 36] were classified as high risk of bias at the flow and timing domain of risk of bias, in which no studies blinded cross-verification and observer calibration before data collection and drop-out of the study sample were seen. Moreover, another seven studies [16, 17, 30, 31, 34, 35, 37] were not undergone with blinded cross-verification and observer calibration “before data collection” (some studies calculated agreement after data collection). Thus, the reviewers identified these studies as unclear risk of bias.

Six studies [21, 30, 32, 34, 36, 37] reported the diagnostic accuracy of CBCT in detecting MB2. Two [33, 35] reported the diagnostic effectiveness of CBCT for the detection of second canal anatomy in permanent mandibular molars. Three [16, 17, 31] investigated second canal anatomy of the maxillary and mandibular premolars. One [29] was identified as the diagnostic study of CBCT in detecting second canal anatomy of permanent mandibular canines.

Micro-CT was used as the reference standard by five studies [17, 21, 32, 33, 36] of second canal anatomy, staining and clearing as the reference standard by three included studies [29, 31, 35], and root sectioning by four studies [16, 30, 34, 37] of meta-analysis.

Three studies [16, 29, 31] reported a prevalence of second canal anatomy less than or equal to 30%. Six studies [17, 32–36] reported the prevalence of the second canal to be between more than 30% to less than 70%. Three studies [21, 30, 37] observed the prevalence of second canal to be more than or equal to 70%.

Ten studies [16, 17, 21, 29–31, 34–37] on the second canal were set as diagnostic cohort studies and agreement studies matched with cohort design and two [32, 33] of the second canal were diagnostic case-control studies.

Five studies [29, 32, 34, 36, 37] of the second canal reported a single spectrum of second canal and seven included studies [16, 17, 21, 30, 31, 33, 35] reported a multiple spectrum of second canal anatomy.

A summary of the findings of the general characteristics of this meta-analysis is listed in Table 1.

**Figure 4:**
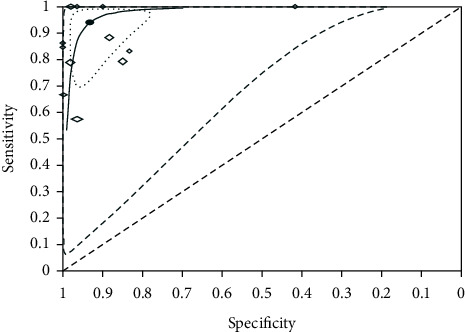
Summary receiver operating characteristic (SROC) curve of CBCT for detection of second canal in permanent teeth.

Pooled estimates of sensitivity and specificity for detection of second canal anatomy of permanent teeth were calculated and described (see Table 2). The summary receiver operating characteristic (SROC) curve of CBCT for detection of second canal in permanent teeth was drawn (see Figure 4).

**Figure 5:**
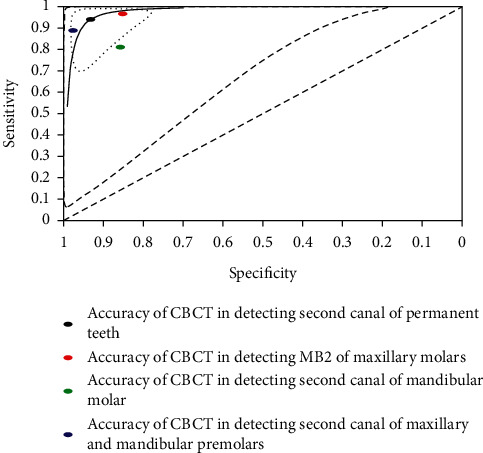
Summary receiver operating characteristic (SROC) curve of CBCT for detection of different types of permanent teeth except for mandibular canines.

**Figure 6:**
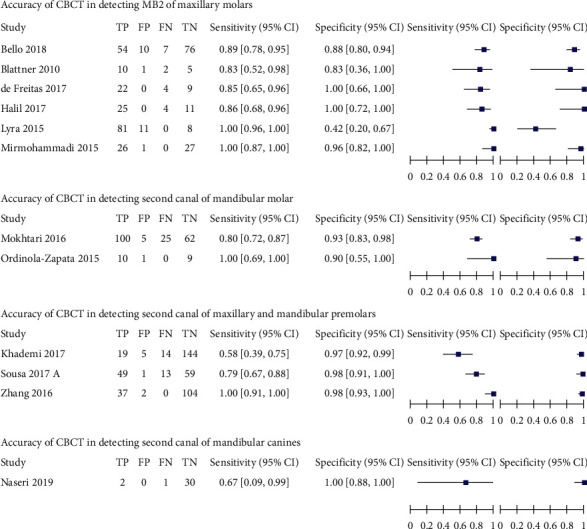
Forest plot of CBCT for detection of second canal in different types of permanent teeth.

Pooled estimates of sensitivity and specificity of CBCT for detection of different types of permanent teeth except for mandibular canine, summary receiver operating characteristic (SROC) curve of CBCT for detection of different types of permanent teeth, except mandibular canines, and forest plot of CBCT for detection of second canal in different types of permanent teeth were calculated and drawn (see Table 3, Figure 5 and Figure 6).

**Figure 7:**
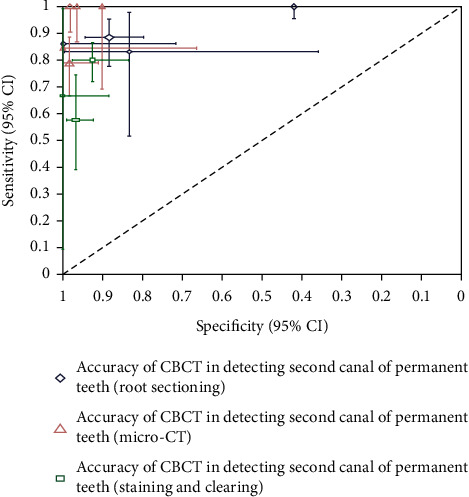
Crosshairs plot of CBCT for detection of second canal according to types of reference standard.

Pooled estimates of sensitivity and specificity of CBCT for detection of second canal according to types of the reference standard and crosshairs plot of CBCT for detection of second canal according to types of reference standard were analyzed and sketched (see Table 4 and Figure 7).

**Figure 8:**
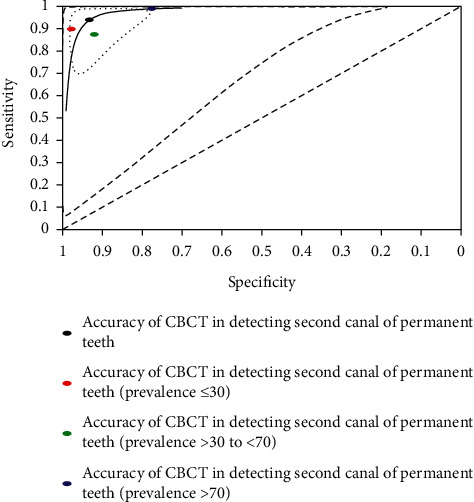
Summary receiver operating characteristic (SROC) curve of CBCT for detection of second canal according to level of prevalence.

Pooled estimates of sensitivity and specificity of CBCT for detection of second canal according to the level of prevalence and summary receiver operating characteristic (SROC) curve of CBCT for detection of second canal according to the level of prevalence were investigated and illustrated (see Table 5 and Figure 8).

**Figure 9:**
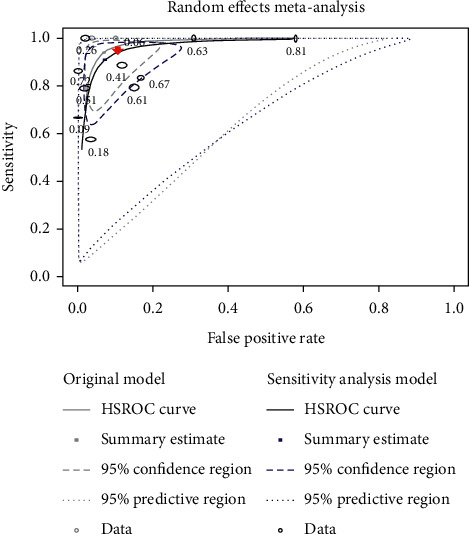
Summary receiver operating characteristic (SROC) curve of CBCT for detection of second canal after exclusion of diagnostic case-control studies (^*∗*^red-filled arrow indicates the drop of sensitivity).

Pooled estimates of sensitivity and specificity of CBCT for detection of second canal before and after exclusion of diagnostic case-control studies and summary receiver operating characteristic (SROC) Curve of CBCT for detection of second canal after exclusion of diagnostic case-control studies were computed and illustrated (see Table 6 and Figure 9).

**Figure 10:**
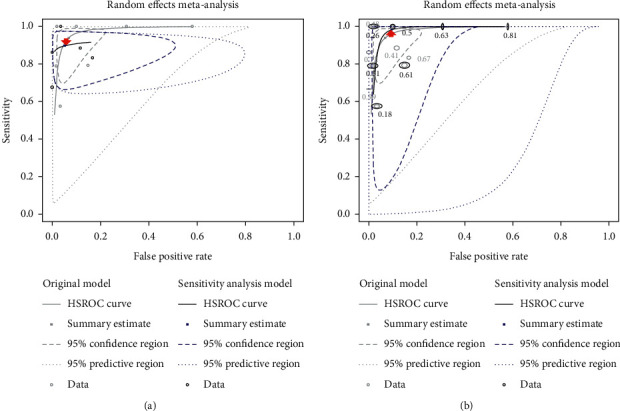
Summary receiver operating characteristic (SROC) curves of CBCT for detection of single spectrum and multiple spectrum of second canal anatomy of permanent teeth (^*∗*^red-filled arrows indicate the drop and uphill of sensitivity). (a) Single spectrum of second canal. (b) Multiple spectrum of second canal.

**Figure 11:**
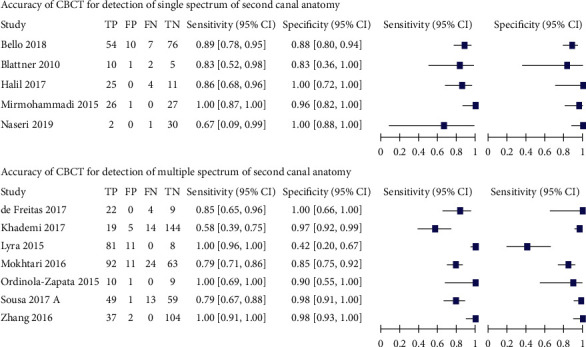
Forest plot of CBCT for detection of single and multiple spectrum of second canal anatomy in permanent teeth.

Pooled estimates of sensitivity and specificity of CBCT for detection of single and multiple spectrum of second canal anatomy in permanent teeth, summary receiver operating characteristic (SROC) curves of CBCT for detection of single spectrum and multiple spectrum of second canal anatomy of permanent teeth, and forest plot of CBCT for detection of single and multiple spectrum of second canal anatomy in permanent teeth were estimated and configured (see Table 7 and Figures 10 and 11).

**Figure 12:**
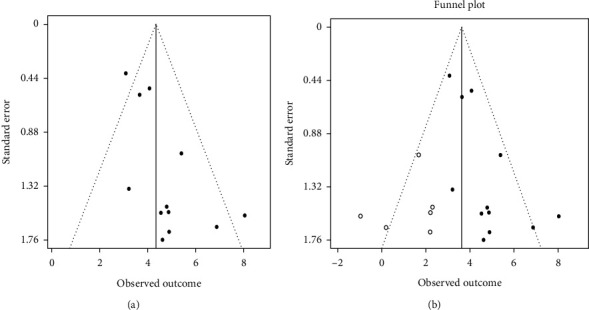
Unadjusted and adjusted funnel plots by trim-and-fill method (6 opened circles in second figure represent filled studies). (a) Unadjusted funnel plot. (b) Adjusted funnel plot by the trim-and-fill method.

There were two funnel plots; one presented the original “asymmetrical” funnel plot of which the bottom of the left side lacked the included studies and another one, the adjusted funnel plot by trim-and-fill method (see Figure 12). In the second figure, the opened circles represented the studies that needed to be filled to achieve funnel plot symmetry.

After adjustment with the trim-and-fill method, 47% reduction of pooled DOR (diagnostic odds ratio) was detected (see Table 8).

## 4. Discussion

### 4.1. Summary of Main Findings

The included studies permitted us to evaluate the diagnostic accuracy of CBCT for the detection of second canal. Unfortunately, summarizing the effectiveness of CBCT in detecting the second canal anatomy of permanent mandibular canine was not possible to estimate due to sparse data. However, the narrative interpretation of the forest plot was possible. The lack of eligible studies was encountered for the remaining types of teeth, especially permanent mandibular incisors.

Estimated summary values of 12 included studies of CBCT for detection of second canal anatomy in permanent teeth were 94% sensitivity (95% CI: 80.7% to 98.3%) and 93.1% specificity (95% CI: 84.4% to 97.2%). Then, we assigned these pooled sensitivity and specificity values to 1000 roots of a hypothetical cohort of permanent teeth with a 46% pooled prevalence of second canals (Table 2).

When the prevalence of second canal was 46% (Second Canal = 460) in 1000 roots of a hypothetical cohort of permanent teeth (Table 2), 28 roots with second canal were not detected (false negative) and second canals were wrongly found in 38 roots with no second canal anatomy in CBCT (false positive).

The patients with false-negative results may suffer from the negative consequences of undertreatment endodontics. The delay in timely treatment can lead to more invasive intervention (surgical endodontics) and threatening the survival of the tooth (extraction). On the other hand, those with false-positive results may be overtreated with an unnecessarily high cost and long treatment time (long-term follow-up).

Based on the Summary Receiver Operating Characteristic (SROC) curve of CBCT for detection of second canal in permanent teeth (Figure 4), 12 studies, SROC curve, and summary point of second canal were aggregated to the upper left-hand corner, which indicated the precision of the pooled estimate. The narrower ellipse was the confidence region in which the summary point and SROC curve were positioned. The effect sizes of the future studies were estimated to be within the confidence region (within-study heterogeneity). The wider one, prediction region, pointed out the extent of between-study heterogeneity. We affirmed that the accuracy of CBCT was informative to detect the second canal although the huge area of between-study heterogeneity was present because confidence and prediction regions were located at thw left hand of and above the diagonal line. The uninformative test is represented below the diagonal line. With the ellipses becoming narrower, the findings are more accurate and between-study and within-study heterogeneity are less.

MB2 was the most prone to be detected in CBCT (Table 3), comprising a sensitivity of 96.6% (95% CI: 82.5% to 99.4%) out of other permanent teeth included in this review, followed by a sensitivity of 88.8% (95% CI: 40.7% to 98.9%) of maxillary and mandibular premolars. Second canal of mandibular molar was less vulnerable to be detected in CBCT than the former two, having the estimated sensitivity of 81% (95% CI: 73.2% to 86.9%).

This means that MB2 was correctly detected nearly in eight more roots than second canals of maxillary and mandibular premolars and 15 more roots than those of mandibular molars out of every 100 roots with second canals. Second canal of maxillary and mandibular premolars was correctly detected in nearly eight more roots than those of mandibular molars out of every 100 roots with second canals.

The actual absence of second canal in maxillary and mandibular premolars was mostly detected in CBCT, estimating 97.6% specificity (95% CI: 94.8% to 98.9%). This was trailed by 85.7% specificity (95% CI: 76.5% to 91.7%) of second canal of mandibular molar and 85.1% (95% CI: 65.2% to 94.6%) of MB2. This means that mandibular molar and maxillary molars resulted in 12 additional false-positive diagnoses compared with maxillary and mandibular premolars. The scenario was confirmed in the SROC curve (Figure 5).

However, the pooled estimates of sensitivity and specificity of permanent mandibular canine were not allowed to be calculated by MetaDTA because at least two studies were needed to be pooled in this software. The summary estimates of sensitivity and specificity of CBCT for detection of second canal in permanent mandibular canines were narratively interpreted, 67% (95% CI: 9% to 99%) and 100% (95% CI: 88% to 100%) in forest plot (Figure 6). in spite of a large amount of uncertainty (wide confidence interval) being present in sensitivity estimate.

The pooled estimate of the sensitivity of CBCT for detection of second canal was 100% (95% CI: 1.7% to 100%) when micro-CT was the reference standard (Table 4). However, there was an imprecise estimate because of the extreme confidence interval. Then, the second tier was 93.8% sensitivity (95% CI: 77.3% to 98.6%) when the reference standard was root sectioning. The sensitivity dropped to 66.8% (95% CI: 48% to 81.4%) after the staining and clearing method was used as the reference standard. On the other hand, the value of specificity was the slightest, 82.9% (95% CI: 57.6% to 94.5%) if root sectioning was the reference standard. The specificities for both micro-CT and staining and clearing (reference standards) were 95.6% (95% CI: 85.5% to 98.7%) and 95.2% (95% CI: 83.9% to 98.7%). As a result, the sensitivity and specificity of CBCT were highest to detect the second canal when micro-CT was used as the reference standard. The crosshairs plot confirmed this evidence (Figure 7).

The summary sensitivity of CBCT increased to 99.1% (95% CI: 65.9% to 100%) when the prevalence of second canal was more than or equal to 70% (Table 5). This was followed by 89.8% sensitivity (95% CI: 34.1% to 99.3%) with a prevalence of less than or equal to 30% (lowest prevalence) and 87.5% sensitivity (95% CI: 76.2% to 93.8%) with more than 30% to less than 70% prevalence of second canal (moderate prevalence). Conversely, the specificity of CBCT was highest, 97.6% (95% CI: 94.7% to 99%), with the lowest prevalence of second canal. This was trailed by 91.9% specificity (95% CI: 84.8% to 95.9%) of the moderate prevalence. The lowest specificity, 77.5% (95% CI: 31.9% to 96.2%), was investigated in the highest prevalence of second canal. As a result, the trade-off of sensitivity and specificity of CBCT switched between the highest and lowest prevalence of second canal anatomy. The situation was affirmed with a SROC curve (Figure 8).

After exclusion of case-control study (Table 6), sensitivity of CBCT for detection of second canal anatomy downgraded from 94% (95% CI: 80.7% to 98.3%) to 90.9% (95% CI: 76.1% to 96.9%) and specificity from 93.1% (95% CI: 84.4% to 97.2%) to 92.9% (95% CI: 82% to 97.4%). The sensitivity of excluded case-control design was 3% less than the pooled estimate although the tiny fall of the specificity being present. This finding was secured by the sensitivity analysis SROC plot (Figure 9) in which the blue-lined ellipses represented sensitivity analysis and the faded-lined being the original summary estimate. Red-filled arrow indicated the drop of summary sensitivity after exclusion of case-control studies.

When the included studies used single spectrum of second canal, the summarized sensitivity was 89.7% (95% CI: 81.8% to 94.5%), which exaggerated to 98.3% (95% CI: 64.7% to 99.9%) with the studies having multiple spectrum of second canal (Table 7). On the other hand, the pooled specificity 90.5% (95% CI: 74.3% to 96.9%) was identified in multiple spectrum second canal anatomy studies, uprising to 94.9% (95% CI: 83.1% to 98.6%) in single spectrum studies.

The trade-off of sensitivity and specificity interfaced between single spectrum and multiple spectrum of second canal anatomy (Figure 10). The faded ellipses represented summary estimates and the blue-lined ellipses resulting from sensitivity analyses of single spectrum and multiple spectrum second canal anatomy. In this figure, a dive of pooled estimate and SROC curve was seen in single spectrum. Although the multiple spectrum has pulled SROC curve upwards, there were huge areas of confidence and prediction regions. The lesser overlap and greater dispersion of confidence intervals were counterchecked in the forest plot of multiple spectrum compared to single spectrum (Figure 11).

### 4.2. Strength and Limitations of the Review

The main strength of this review was that rigorous literature search and aggressive statistical models were used. The literature search was performed using seven databases in this review. The grey literature and non-PubMed-indexed articles were adequately found in Google Scholar, ProQuest, and Scopus.

The bivariate method is the appropriate hierarchical meta-analysis, which directly depends on the paired estimate, sensitivity, and specificity [27]. In contrast to this model, some researchers used the Moses–Littenberg method. This model was based on SROC, being a univariate method, and more importantly, not being able to perform between-study heterogeneity exactly. Meta-DiSc software (http://www.hrc.es/investigacion/metadisc.html), freely available online, was fitted in this model. RevMan 5.3 used in this review was also grounded from the Moses–Littenberg method. As a result, the data from RevMan were adapted into web-based MetaDTA in this review. So, the parameter estimates and standard errors were calculated in MetaDTA software, which allows a bivariate model. Bivariate model, in which summary estimates and standard error can be calculated, estimates within-study and between-study heterogeneity more accurately than the Moses–Littenberg method.

The sensitivities and specificities, calculated from this model, pictured the clinical utilization of CBCT in detecting the missed canal. If the second canal in permanent teeth can be detected prior to endodontic treatment, then general dental practitioners and endodontists can avoid unnecessary endodontic treatment failure, undertake timely referral, and can reduce excessive costs and disease burden for the patients. Furthermore, these oral healthcare professionals can justify the diagnosis according to the findings of the review.

Root canal anatomy has been investigated through a series of systematic reviews [3–6] of CBCT studies. Second root canal was dominant in mandibular anterior of male gender [4], third canal, Vertucci's Type VΙΙΙ not seen in mandibular incisors [3], and pooled estimate of bilateral symmetry of second canal being more prevalent in mandibular lateral incisors than in central in some populations [5]. However, the DTA studies of CBCT for detection of second canal in mandibular incisors were not eligible in this review. Second canal configuration of the mesial root of mandibular first molars was 94% in one systematic review [6]. The summary estimate of 21 studies found that 60% prevalence of MB2 was seen in maxillary first molars [9].

However, the DTA (Diagnostic Test Accuracy) review of CBCT in detecting the root canal anatomy is needed for today's dentistry. Failure to detect root canal anatomy correctly in CBCT was one of the known pieces of evidences. C-shape Vertucci's Type Ι root canal (one canal and one foramen) were misdiagnosed in CBCT as Type ΙΙΙ (second canal) in permanent mandibular first premolar (false positive) [16]. Additionally, Type Ι of the premolar was misdiagnosed as Type VΙ (second canal) in CBCT scan (false positive) [17]. Moreover, Types ΙV and V (second canal) were misinterpreted as Type Ι in CBCT scanning human premolars (false negative) [17]. Associated periapical pathology was parallel with the missed root canal anatomy [15]. The fourth most common case that needed endodontic retreatment was missed second canal [14]. The current DTA review of CBCT for detection of second canal anatomy tried to summarize the current evidences.

The direct comparisons were not applied to detect the diagnostic accuracy of CBCT in different types of teeth; however, a growing body of evidence of DTA studies of CBCT on the individual group of teeth existed during the decade [16, 29, 30, 33, 38]. The most common were the effectiveness of CBCT in detecting MB2 of maxillary molars [21, 30, 32, 34, 36, 37], root canal anatomy of mandibular molars [33, 35], the maxillary and mandibular premolars [16, 17, 31], mandibular canine [29], and mandibular incisors [38]. This review aimed to summarize the available evidence and tackled the indirect comparisons among sensitivities and specificities of CBCT to the different types of teeth.

Micro-CT was the current gold standard method in investigating root canal anatomy. On the one hand, it cannot be used in clinical practice because it has the following disadvantages: being an in vitro method and expensive and having a higher radiation dose than CBCT, taking a long time, and needing a high learning curve to handle it [13]. Moreover, the agreement between CBCT and different reference standards varied across the studies [17, 30, 35]. This review highlighted the variation in sensitivity and specificity of CBCT to detect second canal coupled with the choice of reference standards.

The DTA systematic review [39] of the DTA studies in dentistry tried to point out that the increase in sensitivity of the index test was prone to be associated with a disease prevalence of the study population. This scenario was confirmed by the methodological review [40]. This systematic review confirmed the findings of old evidence.

Usage of diagnostic case-control study inflated the sensitivity of the index test. This meta-analysis stated that overestimation of the findings was influenced by the methodological flaws and design features of individual DTA study. The research methodologists mostly preferred diagnostic cohort studies [40].

Single spectrum [29, 32, 34, 36, 37] and multiple spectrum [16, 17, 21, 30, 31, 33, 35] of second canal were used in the included studies. The researchers of DTA studies of root canal anatomy need to be informed that the spectrum of second canal adversely controlled the estimates. This effect was defined as spectrum effect or spectrum bias [40]. Additionally, the methodological review pointed out that lack of presentation of patient spectrum led to overestimation of overall accuracy of DTA studies [40].

Types of teeth, types of reference standard tests, level of prevalence, types of study design, and types of spectrum of second canal were the effect modifiers on the pathway between the diagnostic accuracy of CBCT (intervention) and the second canal anatomy (outcome). So, the multiple subgroup and sensitivity analyses of the review neutralized the pooled estimates of this review. The trade-off between the sensitivities and specificities was detected in case of the level of prevalence and spectrum of second canal.

A small sample size was usually detected in DTA studies of CBCT for detection of root canal anatomy of permanent teeth. The small sample was disproportionately divided into four subgroups of results: true positive, false negative, false positive, and true negative. This, in turn, reduced the sample size of the individual subgroup, resulting in jeopardizing the external validity and generalizability of the study.

The resolution of CBCT depends on processing time and more precisely voxel size. The smaller the voxel size and the more the processing time, the higher the spatial resolution. The field of view was oriented to reduce the radiated area [21]. The narrower the field of view, the lower the radiation dose to the unnecessary surrounding area. The above-mentioned factors, in addition to milliampere, slice thickness, and kilovolt, can possibly have clinical heterogeneity.

Moreover, there were no eligible DTA studies of CBCT for mandibular incisors and no adequate evidence to summarize the accuracy of CBCT for detection of second canal anatomy of mandibular canines. These limitations made the review imperfect. The reviewers stated that this type of DTA study needs to be performed more frequently the optimal research methodology and adequate sample size.

### 4.3. Methodological Flaws and Risk of Bias

In the patient selection domain, all of the included studies used a convenient sampling method. Lack of randomization may lead to the overestimation of the diagnostic study. To solve the problem of deficient randomization, some reviewers in dentistry suggested that the patient who needs tooth extraction can be randomized to be scanned by index test [41].

Another problem was the usage of single spectrum of second canal, which may lead to “limited challenge bias” [40] in which constricted categories of a sample, such as only Vertucci's Type Ι (true negative) and Type ΙΙΙ (true-positive second canal), were used. Extreme values of Type VΙ, Type VΙΙ, Type ΙΧ, and other types were excluded, known as “spectrum effect” [40], which can influence the findings of the test. So, the methodologists preferred are “consecutive series” [40] in DTA studies, which are less prone to bias.

The prevalence of second canals was frequently detected in MB2 of maxillary molars. The pretest probability of occurrence of second canal can increase the sensitivity of the test. This is known as “context bias” [40]. This bias, the liability of observer of index test to think test findings to be positive more commonly in settings with higher disease prevalence, may overestimate sensitivity of the test.

The index test result being interpreted with the knowledge of finding of reference standard, and vice versa may cause “diagnostic review bias” and “test review bias” [40]. Lack of calibration in some studies may lead to “observer variability” [40]. So, the research methodologists preferred blinded and independent assessment of index test and reference standard, and observer calibration before data collection [40].

### 4.4. Applicability of the Findings

All of the included studies were in vitro studies. The finding from this type of study cannot be completely favorable to the real-life environment because they were controlled experiments, which may jeopardize the external validity of the selected studies.

Some of the included studies did not use an artificial environment [29–31, 35]. Simulating the oral structures, such as PDL, bone, and gingiva, around the sampled teeth was favorable in some selected studies [16, 17, 21, 32–34, 36, 37]. However, this situation cannot be totally applied to clinical settings. Calcification, root-filled teeth, motion artifacts, and beam hardening artifacts during CBCT scanning can be difficult to create in the artificial environment. Pig mandible, human dry mandible, condensation silicone, equal parts of gypsum and rice flour, and POP were used in the included studies [16, 17, 21, 32–34, 36, 37]. However, clearly understanding which model was the most applicable to the current clinical situation is challenging.

Staining and clearing [24] and root sectioning were the historical gold standards. Sometimes, man-made errors resulting from these methods cannot be overcome. As a result, micro-CT is suggested to be applied as the gold standard for the current research scenario.

### 4.5. Publication Bias

Reviewers investigated that the small studies involved in this meta-analysis had a large effect, which was confirmed by “funnel plot asymmetry” and six additional studies were needed to be filled for its symmetry (Figure 12). In other words, publication bias was present in this study. Overestimation of the pooled effect size can occur due to the large effect of small studies.

Before trim-and-fill adjustment, DOR (Diagnostic Odds Ratio) was 78. This meant that 78 roots with second canals were accurately detected in CBCT (True Positive) when the second canal was also wrongly seen in 1 root with none of this anatomy in CBCT scan (false positive). After the adjustment, DOR declined to 37. 47% reduction of unadjusted DOR was seen (Table 8). This seemed to be overestimation of the summary estimate of this review. “DOR = 1” means uninformative test.

However, DOR was used only for publication bias method and not for summary estimate of this meta-analysis because the similar DOR may result from different mixtures of sensitivity and specificity [28].

### 4.6. Direction of Future Study

In this review, the reviewers directed the outcome of research interest to the clinical significance of root canal anatomy and the presence or absence of second canal. In reality, the clinical importance of different root canal anatomy and the variety of Vertucci's types should also be considered as a research interest. In this scenario, the sensitivity and specificity may be prone to be lower than those in the findings of this review. Additionally, special attention should be paid to the fact that the DTA (Diagnostic Test Accuracy) review of specific intricate root canal anatomy needs to be tackled in future research.

This meta-analysis aimed to choose the commonly used CBCT scanner when multiple scanners were comparatively used in the same study. Otherwise, some clinicians would need to know the effectiveness of different types of CBCT scanners. As a result, a network meta-analysis is a suitable option when the multiple and indirect comparisons of different CBCT scanners are intended to be performed.

## 5. Conclusion

The summary accuracy of CBCT for detection of second canal anatomy is important for clinical decision-making of general dental practitioners and endodontists. The pooled estimates of the accuracy of CBCT were 94% sensitivity and 93.1% specificity for second canal of permanent teeth. So, CBCT is definitely informative to detect second canal. The clinicians should keep in mind that the accuracy can vary according to different types of teeth, the prevalence of second canal of different populations, and the spectrum of second canal anatomy. Research implications suggest that sensitivity and specificity of CBCT can change with different types of reference standards used in DTA studies and types of study design. The publication bias also pointed out that the small sampled studies of the meta-analysis had a large effect size. The reviewers postulated that the above-mentioned factors may lead to overestimation of accuracy of CBCT for detection of second canal of permanent teeth. The researchers should be informed that the number of DTA studies of CBCT investigating second canal anatomy and intricate root canal anatomy are needed to be intervened in vivo, especially in index test, diagnostic cohort study design, optimal research methodology, and adequate sample size.

## Figures and Tables

**Table 1:** Summary of findings of general characteristics of diagnostic accuracy of CBCT for detection of second canal of the root canal system of permanent teeth.

Question	What is the diagnostic accuracy of CBCT for detection of second canal of permanent teeth?
Population	The permanent teeth which had no root anomalies and no calcification
Index test	CBCT
Target condition	Second canal of root canal system of permanent teeth
Reference standard	Micro-CT (or) root sectioning (or) staining and clearing
Action	If second canal in permanent teeth can be detected prior to endodontic treatment, then general dental practitioners and endodontists can avoid unnecessary endodontic treatment failure, undertake timely referral, and can reduce high cost for the patients
Diagnostic stage	Aimed at general dental practitioners and endodontists or oral radiologists investigating the patients who need to undergo root canal therapy and retreatment or surgical endodontics
Quality of evidence	12 studies supporting data for meta-analysis
	1084 roots involved in meta-analysis
	10 diagnostic cohort studies and two diagnostic case-control studies involved in meta-analysis
	1 study investigated root-filled teeth
	46% pooled prevalence of second canal of all included studies

**Table 2:** Pooled estimates of sensitivity and specificity of CBCT for detection of second canal anatomy of permanent teeth.

Root canal anatomy	Pooled estimates of all second canal (MB2, maxillary and mandibular premolars, mandibular molars, and mandibular canines)
Pooled prevalence (%)	46

Sensitivity (CI)	94% (80.7%–98.3%)

Specificity (CI)	93.1% (84.4%–97.2%) 1000 (second canal = 460)

Hypothetical cohort of 1000 roots of permanent teeth with 46% prevalence of second canal	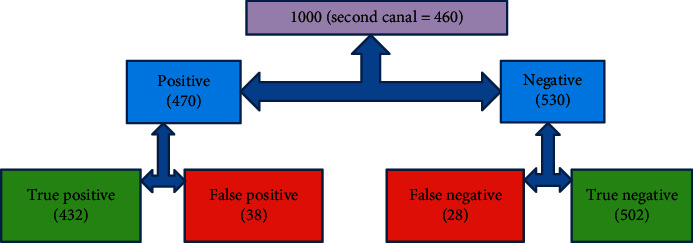

MB2: second mesiobuccal canal of permanent maxillary first and second molars; %: percentage; CI: confidence interval.

**Table 3:** Sensitivity and specificity of CBCT for detection of different types of permanent teeth except for mandibular canine.

Second canal	MB2	Maxillary and mandibular premolars	Mandibular molars
Pooled prevalence (%)^*∗*^	59	29	63
Sensitivity (95% CI)	96.6% (82.5%–99.4%)	88.8% (40.7%–98.9%)	81% (73.2%–86.9%)
Specificity (95% CI)	85.1% (65.2%–94.6%)	97.6% (94.8%–98.9%)	85.7% (76.5%–91.7%)

MB2: second mesiobuccal canal of permanent maxillary first and second molars: %: percentage; CI: confidence interval; ^*∗*^pooled sensitivity and specificity of mandibular canine cannot be estimated because MetaDTA software allows at least two studies to undergo meta-analysis.

**Table 4:** Sensitivity and specificity of CBCT for detection of second canal according to types of the reference standard.

Types of reference standard	Micro-CT	Root sectioning	Staining and clearing
Sensitivity (95% CI)	100% (1.7%–100%)^*∗*^	93.8% (77.3%–98.6%)	66.8% (48%–81.4%)
Specificity (95% CI)	95.6% (85.5%–98.7%)	82.9% (57.6%–94.5%)	95.2% (83.9%–98.7%)

%: percentage; CI: confidence interval; ^*∗*^imprecision of the estimate.

**Table 5:** Sensitivity and specificity of CBCT for detection of second canal according to level of prevalence.

Level of prevalence (%)	≤30	>30% to <70	≥70
Sensitivity (95% CI)	89.8% (34.1%–99.3%)	87.5% (76.2%–93.8%)	99.1% (65.9%–100%)
Specificity (95% CI)	97.6% (94.7%–99%)	91.9% (84.8%–95.9%)	77.5% (31.9%–96.2%)

%: percentage; CI: confidence interval.

**Table 6:** Sensitivity and specificity of CBCT for detection of second canal before and after exclusion of diagnostic case-control studies.

	Before exclusion of diagnostic case-control studies	After exclusion of diagnostic case-control studies
Sensitivity (95% CI)	94% (80.7%–98.3%)	90.9% (76.1%–96.9%)
Specificity (95% CI)	93.1% (84.4%–97.2%)	92.9% (82%–97.4%)

%: percentage; CI: confidence interval.

**Table 7:** Sensitivity and specificity of CBCT for detection of single and multiple spectrum of second canal anatomy in permanent teeth.

	Single spectrum of second canal	Multiple spectrum of second canal
Sensitivity (95% CI)	89.7% (81.8%–94.5%)	98.3% (64.7%–99.9%)
Specificity (95% CI)	94.9% (83.1%–98.6%)	90.5% (74.3%–96.9%)

%: percentage; CI: confidence interval.

**Table 8:** Unadjusted and adjusted diagnostic odds ratios (DOR) with 95% confidence intervals after trim-and-fill method.

Unadjusted DOR (95% CI)	Adjusted DOR (95% CI)
78.06 (36.65–166.26)	37.71 (17.29–82.27)

## Data Availability

The data in this study can be found in the following link: https://doi.org/10.17632/j3k8j58bf7.1.
